# Designing a Standardized and Sustainable Training Framework for Healthcare Workforce Capacity Building in Punjab, Pakistan

**DOI:** 10.3390/healthcare14193187

**Published:** 2026-09-26

**Authors:** Rubeena Zakar, Gulzar Shah, Ain ul Momina, Bushra Shah, Usman Mahboob, Ruhma Shahzad, Muhammad Anawar Janjua, Mukhtar Hussain Syed, Javeria Saleem, Nabeel Ahmad, Nazoora Manal, Mlaika Zakar

**Affiliations:** 1 Department of Public Health, University of the Punjab, Lahore 54590, Pakistan; rubeenaz.iscs@pu.edu.pk (R.Z.); ruhmabshahzad@gmail.com (R.S.); 2 Jian-Ping Hsu College of Public Health, Georgia Southern University, Statesboro, GA 30458, USA; bs06779@georgiasouthern.edu; 3 Department of Community Medicine, King Edward Medical University, Lahore 54000, Pakistan; ainmomina@gmail.com; 4 Institute of Health Professions Education & Research, Khyber Medical University, Peshawar 25100, Pakistan; usman.mahboob@kmu.edu.pk; 5 Licensing and Accreditation, Punjab Healthcare Commission, Lahore 54000, Pakistan; drmajanjua@gmail.com; 6 General Health Punjab, Lahore 54000, Pakistan; drmukhtarsyed80@gmail.com; 7 Department of Public Health, Green International University, Lahore 53000, Pakistan; javeria.hasan@hotmail.com; 8 Dev Data Link, Islamabad 44000, Pakistan; mphilph@gmail.com; 9 Farooq Hospital, Lahore 54570, Pakistan; nazooramanal@yahoo.com; 10 Department of Medical Education, Lahore Medical and Dental College, Lahore 53400, Pakistan; mlaikasarah347@gmail.com

**Keywords:** health workforce, continuing professional development, capacity building, training framework, health systems strengthening, mixed-methods, Punjab, Pakistan

## Abstract

**Background:** A well-trained healthcare workforce is crucial for effective health service delivery and policy implementation. Despite the Primary and Secondary Healthcare Department’s (P&SHD) efforts to improve physical infrastructure, critical gaps persist in the training system. This study aims to develop a standardized, effective, and sustainable training framework for healthcare professionals. **Methods:** The study was conducted from September 2024 to July 2025 using a convergent mixed-methods design. Responses were received from 26 DHDCs, representing an 83.9% response rate, with the DHDC serving as the unit of analysis. The qualitative component included 33 focus group discussions involving 270 participants, as well as 20 key informant interviews. Findings informed the development of the training framework using Kern’s six-step approach and the Hennessy–Hicks Training Needs Analysis Model. **Results:** Of the reporting DHDCs, 96.2% had a dedicated training hall, 96.2% reported internet access, and 80.8% employed information and communication technology in training delivery. In contrast, institutional and governance capacity was markedly weaker: 16 of 26 centers lacked a documented definition of roles and responsibilities. Qualitative findings further revealed that trainings were frequently not needs-based, participant selection was perceived as influenced by favoritism and political considerations, standardized curricula and trainer criteria were absent, and evaluation was limited to attendance and satisfaction metrics. Thus, physical capacity and system functionality diverged substantially: the binding constraints were governance, coordination, staffing continuity, and financing rather than infrastructure. **Conclusions:** This study underscores the critical need for a standardized, sustainable training framework to strengthen the capacity and performance of healthcare providers.

## 1. Introduction

A well-trained, adequately staffed, and motivated healthcare workforce is the cornerstone of any healthcare system [1]. It is also necessary for efficient service delivery, effective policy implementation, and better health outcomes for the population [1,2]. Without skilled professionals, even the well-funded healthcare systems struggle to meet the needs of their communities [1]. Continuing Professional Development (CPD) and training are essential for healthcare workers to stay up to date with medical advancements and evolving healthcare demands. Research underscores the importance of investing in healthcare education and training programs to equip professionals with the necessary skills and knowledge to address global health challenges [1,2,3]. Investment in healthcare human resources directly influences the quality of care and patient outcomes [3]. CPD also bears on workforce stability: a systematic review of 27 studies, predominantly from the health sector, found associations between access to professional development and the retention of health workers in post [4]. The WHO’s Global Strategy on Human Resources for Health provides a framework for strengthening healthcare workforce capacity, improving health outcomes, and achieving Universal Health Coverage (UHC) [5]. Recent evidence indicates that increasing training volume alone does not reliably change provider practice: a systematic review of strategies to improve healthcare provider performance in low- and middle-income countries found that training delivered in isolation produced only moderate effects [6]. In Pakistan, CPD lacks an agreed national standard, and a recent Delphi study developing CPD standards for allied health professionals found no established local standards, with time and budget constraints identified as principal barriers [7]. Although several studies have examined healthcare workforce training needs in low- and middle-income countries [1,2,3,5], limited evidence exists on integrated, system-level frameworks that combine governance, competency development, monitoring and evaluation, and institutional sustainability. This study contributes to the literature by proposing a context-specific and evidence-informed framework to strengthen in-service training for the healthcare workforce in Punjab, Pakistan.

### 1.1. Context of Punjab

Punjab, the most populous province in Pakistan, operates under two health departments to address the professional development and training needs of its healthcare workforce: the Primary & Secondary Health Department (P&SHD) and the Specialized Health & Medical Education Department (SH&MED). The P&SHD manages primary and secondary healthcare facilities, whereas SH&MED oversees teaching hospitals and medical education. The P&SHD administration operates at the provincial, district, and tehsil levels. The department is headed by the Secretary of P&SHD, who provides policy guidance and strategic direction. It is supported by two directorate generals who manage technical healthcare operations.

At the district level, the District Health Authority (DHA) functions under the Local Government Act, overseeing the administration of primary and secondary healthcare facilities. Each DHA is led by a Chief Executive Officer (CEO) and is headed by its chairman (presently, the Deputy Commissioner of the district is designated as chairman), who oversees the administration of public health programs, Basic Health Units (BHUs), Rural Health Centers (RHCs), Tehsil Headquarters Hospitals (THQH), and District Headquarters Hospitals (DHQH). The DHA is also responsible for ensuring the availability of essential health services, implementing disease control programs, monitoring health indicators, and coordinating district-level responses to public health emergencies. Punjab’s public healthcare system comprises over 4700 medical facilities, including BHUs, RHCs, THQH, DHQH, and teaching hospitals.

### 1.2. Health Indicators of Punjab

Although Punjab outperforms national averages in several health indicators, significant progress is needed to meet national and international targets, particularly those outlined in the Sustainable Development Goals (SDGs). As the largest province, which houses more than 50% of the country’s population, Punjab plays a crucial role in shaping national health outcomes. Key health statistics include a UHC index of 53.9% [8], a maternal mortality ratio of 157 per 100,000 live births, an under-five mortality rate of 85 per 1000 live births, an infant mortality rate of 73 per 1000 live births, an 80% full childhood immunization rate, and a 34% prevalence of stunting among children [9]. These indicators characterize the scale and complexity of service demand placed on the provincial health workforce, and thus the competencies the training system must support; they are not presented as outcomes attributable to training arrangements, and no causal relationship between in-service training provision and these indicators is claimed or tested in this study.

### 1.3. Structure, Functions, and Responsibilities of the Provincial and District Health Development Centre

Recognizing the importance of professional development and the need to ensure accessible, high-quality healthcare through a competent workforce, the P&SHD established a central Provincial Health Development Centre (PHDC) at the provincial level and District Health Development Centers (DHDCs) in 31 districts of the province to facilitate CPD for healthcare professionals. The PHDC operates under the Directorate General Health Services (DGHS), while DHDCs function under the administrative control of DHAs and technical oversight of the PHDC.

Due to scarcity of funds, P&SHD arranges only a limited number of technical training programs, primarily focused on emerging epidemics or disease outbreaks [10]. Vertical programs funded through PC-1 budgets and supported by different development partners organize most of the trainings independently, often bypassing DHAs, PHDC, and DHDCs. This lack of coordination results in fragmented training efforts, duplication, and exclusion of the relevant target audience.

Presently, P&SHD training initiatives lack a centralized coordination mechanism that links department-led training, vertical programs, and development partners. The absence of an annual training plan, clear targets for in-service professionals, and adequately structured response trainings for epidemic outbreaks further weakens the system [10]. Resultantly, the training efforts remain reactive, with limited long-term impact on healthcare service quality and workforce competencies [10].

Recently, P&SHD has made significant efforts to enhance the physical infrastructure of DHDC, such as renovating training halls, upgrading libraries, modernizing office spaces, and essential training-related furniture and equipment to foster a more effective learning environment [10]. Despite these advancements, critical gaps remain in the overall training system. Key issues include the absence of comprehensive training needs assessments, standardized module development, clear criteria for selecting resource persons, and robust monitoring and evaluation mechanisms [10]. Without addressing these gaps, training programs risk failing to achieve their intended impact on healthcare service delivery.

### 1.4. Problem Statement

The training system for Punjab’s public-sector health workforce exhibits four documented deficiencies: training is not preceded by systematic needs assessment; delivery is fragmented across departmental, vertical-programme and development-partner channels without a coordinating mechanism; standardised curricula and criteria for selecting resource persons are absent; and evaluation does not extend beyond attendance and satisfaction [10]. Recent departmental investment has concentrated on physical infrastructure, leaving these systemic determinants unaddressed. The scholarly gap is corresponding: existing work has characterized training needs and evaluated discrete programs, but has not produced an integrated framework specifying governance, needs assessment, curriculum standardization, evaluation, and financing as interdependent components of a single system.

### 1.5. Scope and Aim of Study

This paper is part of a project aimed at providing technical assistance to establish a sustainable, standardized training system for Punjab’s healthcare sector. This initiative aligns with the National Health Support Program (NHSP), particularly with Disbursement Linked Indicators (DLIs) 2 and 10, which focus on enhancing essential health services, improving primary healthcare providers’ performance, and ensuring effective service delivery through integrated training and monitoring.

The proposed in-service training framework may contribute to institutionalizing and standardizing CPD programs, ensuring long-term sustainability and impact. Strengthening training systems has the potential to enhance the financial and technical capacities of local institutions, promoting self-reliance and continuous workforce development. This study was conducted to assess gaps in the existing in-service training system and to develop an evidence-informed framework to strengthen the healthcare workforce capacity in Punjab, Pakistan. The study aims to design a standardized, effective, and sustainable training framework for healthcare professionals. It focuses on:

1. Identifying the professional development needs of Punjab’s healthcare workforce by evaluating existing training programs, identifying gaps, and incorporating best training practices and Pakistan’s global commitments.
2. Designing a structured in-service training framework for healthcare professionals working under P&SHD, Government of Punjab.

The study concerns in-service CPD for the public-sector workforce employed under P&SHD; undergraduate and postgraduate medical education, SH&MED teaching hospitals, and private-sector training fall outside its scope.

## 2. Methods

**Figure 1:**
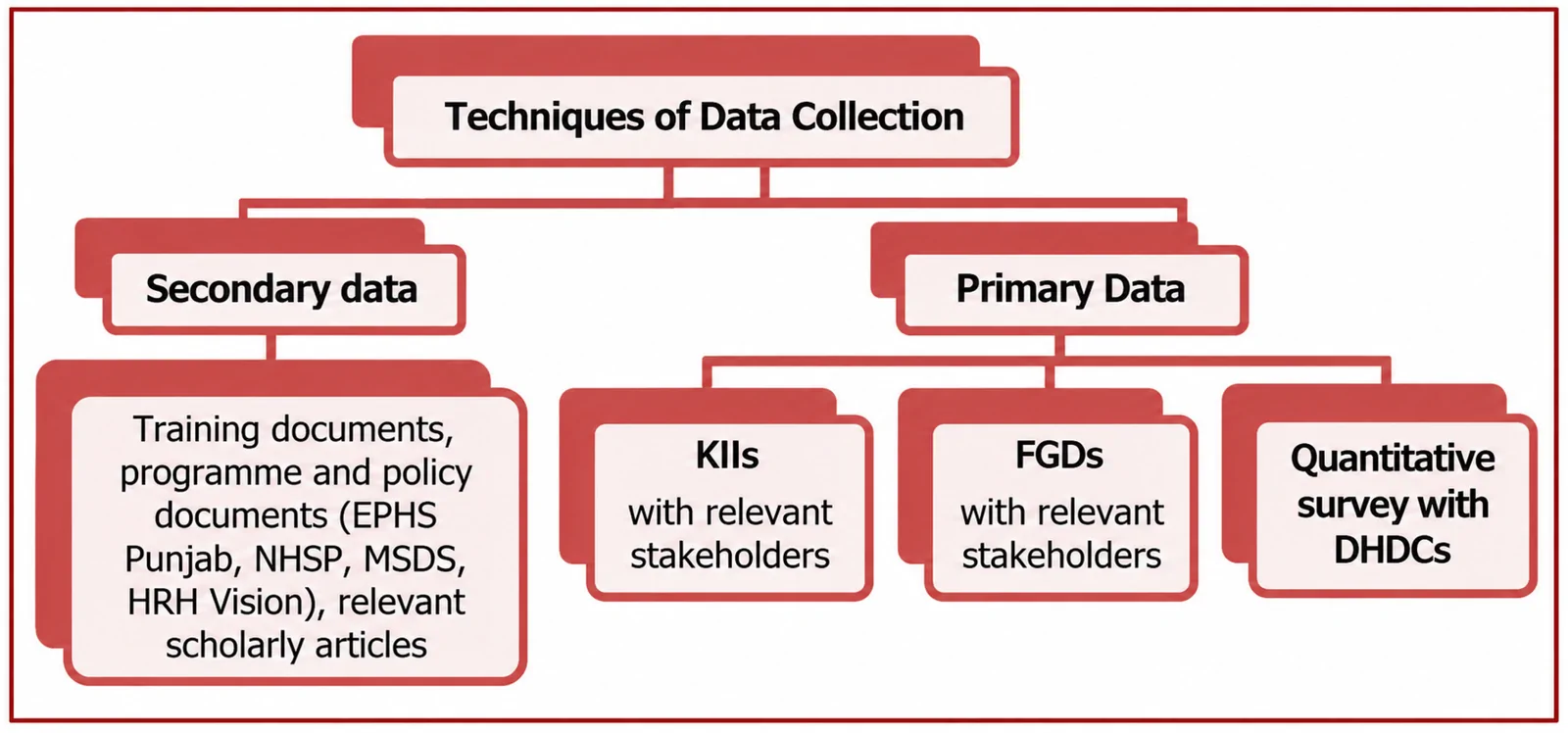
Data sources and data collection methods.

The present study employed a convergent mixed-methods design that integrated quantitative and qualitative data, followed by a framework-development phase. The study was conducted from September 2024 to July 2025. Data were collected using both primary and secondary sources: primary data were collected in parallel through a quantitative survey of DHDCs, Key Informant Interviews (KIIs), and Focus Group Discussions (FGDs). Secondary data comprised program and policy documents and relevant peer-reviewed literature (See Figure 1). Quantitative and qualitative data were analyzed separately and subsequently integrated during interpretation to inform the development of the training framework.

### 2.1. Quantitative Data

The quantitative component consisted of a cross-sectional self-assessment survey conducted across all 31 DHDCs in Punjab, Pakistan. The survey collected primary data on available facilities, infrastructure, and institutional functionality using a structured questionnaire. A modified version of the questionnaire previously employed in an earlier assessment was used [10]. The original instrument had been adapted from a World Health Organization study examining public sector institutional capacity for health workforce governance in the South-East Asia region [11]. Questions that did not align with the objectives of the present assessment were omitted, while additional items specifically addressing healthcare-workforce training were incorporated. These items were informed by the professional-development domains identified in the United States Agency for International Development (USAID) Human Resources for Health Indicator Compendium [12]. The final questionnaire therefore captured information on the availability of training equipment, infrastructure, and internet connectivity, as well as the number of healthcare providers trained at each DHDC. The questionnaire comprised five sections:

1. System-wide factors (framework, roles, responsibilities, institutional linkages);
2. Organizational environment (workload, team structure, supervision, management, and support systems);
3. Individual factors (skills and expertise of health staff);
4. Tools (budget, equipment, and guidelines); and
5. Training capacity (frequency, content, and audience of training).

Additional questions addressed the specific training and professional development needs of the healthcare workforce. The self-assessment questionnaire was sent to all DHDCs across the province via WhatsApp with the administrative support of the PHDC and the P&SHD. Completed questionnaires were returned by 26 DHDCs, corresponding to a response rate of 83.9%; five centers did not respond despite repeated reminders. The unit of analysis was the DHDC rather than the individual healthcare worker. Questionnaires were completed by the heads of the respective DHDCs. To minimize potential reporting bias and to verify the accuracy of responses, supporting documents were requested with each submission. The questionnaire was distributed through official channels of PHDC and P&SHD. Supporting documentation was requested to corroborate institutional responses where available.

### 2.2. Qualitative Data

The qualitative component explored the cultural and contextual factors that shape the effectiveness of the training system and identified adaptations required for local relevance. Insights were drawn from the Competency-Based Training Framework for Service Delivery Staff at the Primary Care Level” [13]. Data were collected through KIIs and FGDs with a range of stakeholders.

Before data collection, a detailed stakeholder-engagement map was developed using the Agency for International Cooperation (GIZ) Capacity Works framework and drawing on prior experience of similar work in other provinces of Pakistan [14,15]. The map identified and categorized relevant stakeholder groups in Punjab according to their roles and responsibilities. These groups comprised government officials from the P&SHD, healthcare providers, and development partners including the World Bank, United Nations agencies, GIZ, and community organizations. The data collection instruments were developed on the basis of an extensive literature review and informal consultations with key stakeholders. The interview guides addressed the current state of the training system, stakeholders’ training needs and expectations, the challenges faced by healthcare providers, and potential areas for improvement.

Eleven districts were purposively selected from three regions of Punjab (south, central, and north). Within each region, a combination of relatively privileged and underprivileged districts was chosen to ensure a balanced perspective and to permit meaningful comparisons across different infrastructural contexts. In total, six privileged and five underprivileged districts were included: Multan and Bahawalpur as privileged and Muzaffargarh as underprivileged from South Punjab; Sialkot and Sahiwal as privileged and Mandi Bahauddin and Jhang as underprivileged from Central Punjab; and Sargodha and Rawalpindi as privileged and Attock and Bhakkar as underprivileged districts from North Punjab.

Thirty-three FGDs were conducted across the selected districts with different stakeholder groups to assess the training needs of multiple healthcare provider cadres. These cadres comprised doctors, paramedics, nurses, outreach program workers, administrative/managerial staff, and developmental partners (see Table 1). The interviews were conducted by a team of trained researchers holding PhD or MPhil degrees in public health and possessing extensive experience in qualitative research.

Participants were recruited through formal institutional channels. Official letters, issued with the support of the PHDC and the P&SHD, were sent to the DHA of each selected district. These letters outlined the purpose of the study, the required workforce cadres and stakeholder categories for each FGD, and the target number of participants sought per group. DHA offices subsequently nominated individual participants in accordance with these specifications.

**Table 2:** Key stakeholders and the number of key information interviews conducted in three regions of Punjab.

Key Stakeholders		South	Central	North	Total
District Heads	DHDCs Head/CEOs	3	4	4	11
Senior officials of P&SHD	Secretary	-	1 (Lahore)	-	1
	DGHS	-	1 (Lahore)	-	1
	Director General (DG) Drug Control	-	1 (Lahore)	-	1
	DG Nursing	-	1 (Lahore)	-	1
	Additional Secretary Technical	-	1 (Lahore)	-	1
Head of PHDC	Program Director (PD)	-	1 (Lahore)	-	1
Heads of Public Universities	Institute of Public Health (IPH) Dean	-	1 (Lahore)	-	1
	University of Health Sciences (UHS)Vice-Chancellor (VC)	-	1 (Lahore)	-	1
	University of the Punjab VC	-	1 (Lahore)	-	1
Total		3	13	4	20

The number of FGDs and KIIs were determined in advance according to the structural coverage required: two FGDs per cadre in each of three regions, together with dedicated groups for development partners and vertical program heads, and KIIs with district-level heads and provincial officials. Adequacy was therefore defined by comprehensive coverage across regions, professional cadres, and levels of governance rather than by monitoring thematic saturation.

Each FGD comprised participants drawn from a single professional cadre. These arrangements enabled training-related issues to be discussed among professional peers rather than in the presence of individuals who held formal authority over the participants. The single cadre design was adopted because several topics of interest, participant selection practices, political influence on training prioritization, and criticism of departmental arrangements are matters on which employees may feel constrained when supervisors are present.

All FGDs and KIIs took place on official premises (DHDCs, DHA offices, or the relevant provincial office) in a private room set aside for the purpose and arranged to ensure participants’ comfort. Only the participants, the interviewer or facilitator, and a note-taker were present. No supervisory, administrative, or departmental personnel from outside the participant group attended any session, and no observers were admitted.

Sessions were conducted bilingually in Urdu and English. Participants were free to use either language, or to switch between them, according to their preference and convenience. Facilitators were fluent in both languages and posed questions in the language being used by the participant or group at that moment. This approach was adopted because the professional vocabulary of the health system in Punjab is predominantly English, whereas everyday narrative expression is more commonly in Urdu; requiring a single language would have constrained participants’ ability to describe their experiences fully.

All sessions were audio-recorded with the prior permission of participants. Permission to record was sought separately from consent to participate, and participants were informed that they could decline recording or ask for the recorder to be stopped at any point without affecting their participation. KIIs lasted between 45 and 60 min; FGDs lasted between 90 and 120 min.

Audio recordings were transcribed and translated into English for analysis by a trained team of researchers holding Ph.D. or M.Phil. degrees. All subsequent analysis was conducted on the English transcripts.

### 2.3. Secondary Data

A structured, extensive desk review was conducted to identify international best practices, policy frameworks, Pakistan’s international commitments, evidence relevant to healthcare workforce training, CPD systems, and established models applicable to the design of a provincial training framework. This was a purposive, structured review of selected sources rather than a systematic or scoping review, and it was not conducted in accordance with PRISMA standards. Documents were identified from three sources. Peer-reviewed literature was retrieved from Scopus using combinations of the search terms “healthcare capacity building”, “health workforce”, “continuing professional development”, “in-service training”, “training needs assessment”, “competency-based training”, “health systems strengthening” and “low- and middle-income countries”, with results restricted to English-language publications. National and provincial policy and program documents were obtained through the P&SHD and the PHDC. International guidance and technical assistance documents were identified through the relevant agencies and the research team’s prior work on comparable assignments in other provinces of Pakistan. Documents were included if they addressed health workforce training systems, CPD, curriculum development, or training evaluation, and if they were either applicable to Pakistan’s policy environment or drawn from settings comparable to Punjab in resource level and system structure.

Documents were reviewed against a common set of categories corresponding to the framework components under consideration (needs assessment, curriculum development, delivery, evaluation, and sustainability). Extracted content was then integrated in the final stage.

### 2.4. Data Analysis

SPSS (Statistical Package for Social Sciences) version 26 was used for quantitative data analysis. Quantitative data from DHDCs were entered into an SPSS sheet and screened for inconsistencies, missing values, and outliers. Descriptive statistics (frequencies, means, and percentages) were computed to summarize key findings.

For qualitative data analysis, thematic analysis was used. We followed Braun & Clarke’s six-step framework [16] for thematic analysis. The audio-recorded qualitative data were translated and transcribed verbatim and reviewed multiple times by two researchers to gain an in-depth understanding. The review helped to identify initial patterns. A systematic coding process was used to label meaningful segments of data. inductive in the first instance. Codes were assigned based on key phrases, recurring words, and emerging patterns. Emerging themes were categorized and refined through iterative analysis. Similar codes were grouped into broader categories (e.g., “challenges in training”, “lack of training”) and categories into themes. Quotations are attributed using a source identifier that combines district, activity type, and cadre of healthcare staff (for example, LKIIPD denotes a key informant interview with a project director in Lahore).

Quantitative and qualitative findings were compared during integration to identify areas of convergence, complementarity, and divergence, while qualitative findings provided contextual explanations for patterns observed in the institutional survey. Two researchers independently coded the qualitative data and compared their coding. Discrepancies in code application and category boundaries were discussed and resolved by consensus. The coding, the developing codebook, and the emerging themes were then reviewed in successive collective meetings of the full research team. In these meetings, coding decisions, category boundaries, and candidate themes were discussed and revised until the final thematic structure was agreed. Themes were subsequently organized against the four levels of the systemic capacity framework, with themes that did not correspond to a level of that framework retained and reported separately. Agreement between coders was established through comparison, discussion, and consensus rather than by calculating a reliability coefficient; no inter-coder reliability statistic was computed.

### 2.5. Data Integration and Development of Training Framework

Findings from the primary (qualitative and quantitative) and secondary data were integrated and triangulated to derive meaningful and comprehensive insights into Punjab’s training needs and existing capabilities. The Hennessy–Hicks [17] Training Needs Analysis (TNA) model was incorporated into Kern’s six-step approach to curriculum development [18] to systematically identify gaps in competencies and training needs. This integrated evidence-based information guided the development of the training framework. The framework was developed by the research team, comprising members with expertise in public health, medical education, health profession education, health system management and provincial health administration. The components of framework were derived from three sources: (1) the deficiencies identified empirically in this study through the institutional survey and the qualitative strand; (2) the requirements imposed by national and provincial policy, principally the NHSP, the EPHS for Punjab and the MSDS; and (3) the structure of established models, principally Kern’s six-step approach to curriculum development [18] for the overall architecture and the Hennessy–Hicks training needs analysis model for the needs assessment component [17].

### 2.6. Validation of Training Framework

Following its development, the proposed training framework was submitted to the PHDC for review. The framework and the accompanying training material were shared with PHDC in advance to allow examination before discussion, and were subsequently presented in person by the research team to the Program Director of the PHDC and the managerial team. The presentation covered the framework in full, its constituent components, the evidence from which each was derived, and the relationships between them, together with the training material developed alongside it.

The framework and accompanying material were endorsed as presented, and no changes to the components, curricular elements or monitoring indicators were required following the review. During the review, the PHDC indicated an intention to pilot two of the training modules. Any such pilot lies outside the scope of the present study, and no pilot findings are reported here.

Review by the PHDC provides an assessment of the framework’s content and institutional relevance by the body that holds technical oversight of CPD across the province, and that would carry operational responsibility for implementation. It does not constitute a feasibility assessment, pilot implementation, or evaluation of effectiveness, none of which was undertaken. The framework is accordingly presented as evidence-informed and institutionally endorsed, but not prospectively evaluated.

## 3. Results

### 3.1. Professional Development Needs of Punjab’s Healthcare Workforce

The data are presented according to the healthcare capacity need assessment pyramid, comprising four levels: (1) system-wide factors, (2) staff and infrastructure, (3) skills, and (4) tools [19].

#### 3.1.1. System-Wide Factors

System-wide factors are at the foundational level of the capacity pyramid, focusing on governance, legal frameworks, roles and responsibilities, and institutional linkages [14].

Most DHDCs (16 of 26) were established between 1997 and 1999. Over half (16 out of 26) lacked a formal document defining roles, responsibilities, and structure. Some DHDCs were involved in budgetary planning (15.4%) and HRH policy management (19.2%). Only 15.4% set regulations and education policies in collaboration with councils and institutions. All DHDCs conducted CPD training for a range of healthcare professionals. About 38.5% focused on HRH policy capacity-building, 23.1% advocated for HRH investments, and 19.2% engaged in labor relations (Table 3). Additional roles included monitoring EPI campaigns, supervising BHUs, and participating in TB, infection control, and awareness programs. There was an inconsistency in system-wide roles, with some DHDCs performing tasks beyond their mandate. One of the study participants opined: “*There is a formal uniform document for all the DHDCs, but as the majority of the DHDCs and their staff are engaged in different tasks of the CEO office, they never bothered to check what their actual duties are*” (LKIIPD).

Another study participant said: *DHDCs and PHDC used to be the epitome of training of healthcare staff when they were initially designed and implemented. I also worked as the PD in one of the DHDCs around 20 years back, but when the DHDCs came under the authority of the CEO from PHDC, the whole structure collapsed, with the majority of the DHDCs now performing the tasks of the CEO and DHA offices (LKIIPD).*

#### 3.1.2. Organizational Environment

This level focuses on workload, team structure, supervision, and management support.

About 88% (23 out of 26) of DHDCs had an organogram, typically structured under a PD with various officers and support staff. The data showed a varied reporting structure: some DHDCs reported to the Director PHDC (2 out of 26) and the CEO DHA (5 out of 26), while most reported to multiple authorities (19 out of 26). There was a lack of coordination among departments and international donors (i.e., UNICEF, JICA). Officials cited inadequate training, notification, and work overload due to a lack of exemptions. One study participant during FGD angrily reported: “I travelled 45 km from my home to the healthcare facility; upon reaching there, I got a call from the CEO’s office for a training at DHDC at 10 am, and while traveling to DHDC, I got calls from my healthcare facility to provide an update of my work” (MFGDOW). Some DHDCs reported weak interdepartmental synergy, leading to delays in planning and implementation.

#### 3.1.3. Individual Factors

This level assesses personnel qualifications, expertise, and experience.

About 7 DHDCs reported a staff member with a Doctorate in Public Health, 15 had an MPH, and 22 had Bachelor’s/tertiary degrees. Around one-fourth of DHDCs mentioned that they had their staff assigned to P&SHD on general duty (6 out of 26; 23.1%). Many DHDCs reported frequent leadership turnover: 54% (14 out of 26) had 3+ project directors (PDs) in five years, 38% (10 out of 26) had 2, and 8% (2 out of 26) had 1, and 34.6% (9 out of 26) of PDs had served for less than a year. 65.2% (15 out of 26) of PDs had ≤3 years of administrative experience; with 38.5% (10 out of 26) holding MBBS and 61.5% (16 out of 26) MBBS with DPH/MPH. Qualitative data found a complaint of frequent transfers, which hinder institutional stability and staff motivation. One of the managerial staff opined: “A person puts effort into building the department’s capacity when he has the assurance that he will serve at the specific top administrative post for at least 5 to 8 years, but we do not have the assurance even for tomorrow” (MFGDAS).

Another study participant said: When a person is fully trained or understands the institutional functioning either through training or experience, the post is changed, ultimately leading to wastage of resources” (MFGDAS). A majority of study participants and DHDCs administrators were of the view that retaining qualified personnel enhances efficiency and reduces administrative burdens.

#### 3.1.4. Tools

This level covers financial, infrastructural, and operational tools.

The study found that DHDC office space averages 6147.87 sqm (range: 437–21,780 sqm). PHDC and DHDCs lacked adequate office space and infrastructure. Around 80.8% (21 out of 26) store data in hybrid formats, while the rest store data on paper only (19.2%; 5 out of 26), and 96.2% (25 out of 26) have internet access. About 69% (18 out of 26; 69.2%) had updated audio–visual equipment, 81% used ICT in training (21 out of 26; 80.8%), and 61.5% had a trainer program (16 out of 26). A majority (25 out of 26; 96.2%) had separate training halls, but 34.6% lacked hostel facilities (12 out of 26). All DHDCs receive funds from the DHA but face budgetary constraints. Training costs (TADAs) were covered in donor-funded programs but not by the health department. Officials reported difficulty in recruiting quality trainers due to inadequate financial incentives. Data found that budget release processes required extensive approvals, delaying fund utilization. Limited public health spending in Punjab restricts training resources and trainer quality. Key officials advocated for direct budget allocation to DHDCs and PHDCs without the finance department’s mediation.

#### 3.1.5. Identifying the Priority Needs of Healthcare and the Political-Economy Landscape

Based on primary and secondary data, as well as stakeholder consultations, 30 priority training areas have been identified. The primary data found that DHDCs prioritized education and capacity building for healthcare workers but overlooked managerial and soft skills. During the year 2024, the key training areas were training for new and existing staff, emerging diseases, TB DOTS, dengue, polio, measles, medico-legal cases, family planning services, newborn resuscitation, smog prevention, nutrition, social mobilization, autism, and women’s health.

Despite the emphasis on training, officials acknowledged the absence of a formal needs assessment mechanism. Trainings were often politically driven, influenced by international funding and political interference. Key issues include political influence in prioritizing training areas, which also affects resource allocation, governance, and participant selection. Many healthcare staff found that existing training was misaligned with on-the-ground requirements.

Officials stressed the need for soft skill and basic life skill training for all healthcare workers. Strengthening managerial skills would help build a competent workforce to provide essential and emergency care, reducing the burden on DHQs and THQs.

#### 3.1.6. Current Training Practices: Clinical/Non-Clinical Priority Areas and Opportunities & Gaps

Training healthcare workers is crucial for equipping HRH with updated skills for preventive, curative, and therapeutic services. In Punjab, various departments and international organizations conduct training, but PHDC remains the only provincial institution that provides multidimensional, inclusive training across all cadres. DHDC focuses on district-level developmental training.

A total of 563 trainings were conducted during the last five years (325 clinical, 148 non-clinical, 90 mixed), by SH&MED (66), P&SHD (478), and external organizations (19). A total of 327 trainings were non-hospital-based, and 236 were hospital-based; 384 required generic, 179 required specialized clinical equipment.

The qualitative data found that training participants were often nominated based on favoritism, limiting opportunities for many, and some professionals received repeated training, while others remained under-skilled. There were inconsistent training packages across districts, considerable variability in assessment mechanisms (pre/post-tests, follow-up), and some reported discrepancies in training experiences. Some study participants held the view that training resources were not always aligned with workforce needs.

Almost all of the study participants recommended developing a standardized training framework. They also suggested direct financial allocation to PHDC and DHDCs for structured implementation and the need for a uniform training package aligned with minimum service delivery standards (MSDS) to ensure consistency. They also emphasized regular monitoring and evaluation mechanisms to assess training impact.

#### 3.1.7. Best Training Practices for Healthcare Workers in Resource-Limited Contexts

International best practices emphasize context-based, need-driven training rather than compliance-based approaches. A sustainable training framework should include the following.

Key recommendations from officials and participants for sustainable training framework development included planning training based on healthcare workers’ job descriptions rather than political priorities. Pre-induction training is essential. One of the study participants opined: “Healthcare workers do not have any orientation when they are initially inducted into their positions” (MFGDAS). There should be clear demarcation of training for administrative cadre as their roles require specialized skills. Some suggest managerial training for all. Almost all study participants opined that PHDC & DHDCs should be the sole authorities managing trainings with intersectoral collaboration. Some officials recommend involving the teaching cadre as well. Some participants suggested that online training can reduce travel time and workload. A portal/app could allow workers to access relevant training at their convenience and maintain a record of completed training.

#### 3.1.8. Significance of Accreditation and Standardization in Training Systems

The secondary data and qualitative interview findings emphasized that a centralized accreditation system is needed to ensure quality, consistency, and competency across training programs. Almost all participants in FGDs were of the view that standardized training packages, clear SOPs, and intersectoral collaboration with teaching institutions would enhance training quality and workforce competency. One of the participants specifically narrated: “I think the biggest loophole is on the technical side. There is a need to develop better modules, standardized trainings, and specifically improved and qualified human resources for making trainings effective (LKIIDVC).”

### 3.2. Systemic Factors Constraining Training Effectiveness

**Figure 2:**
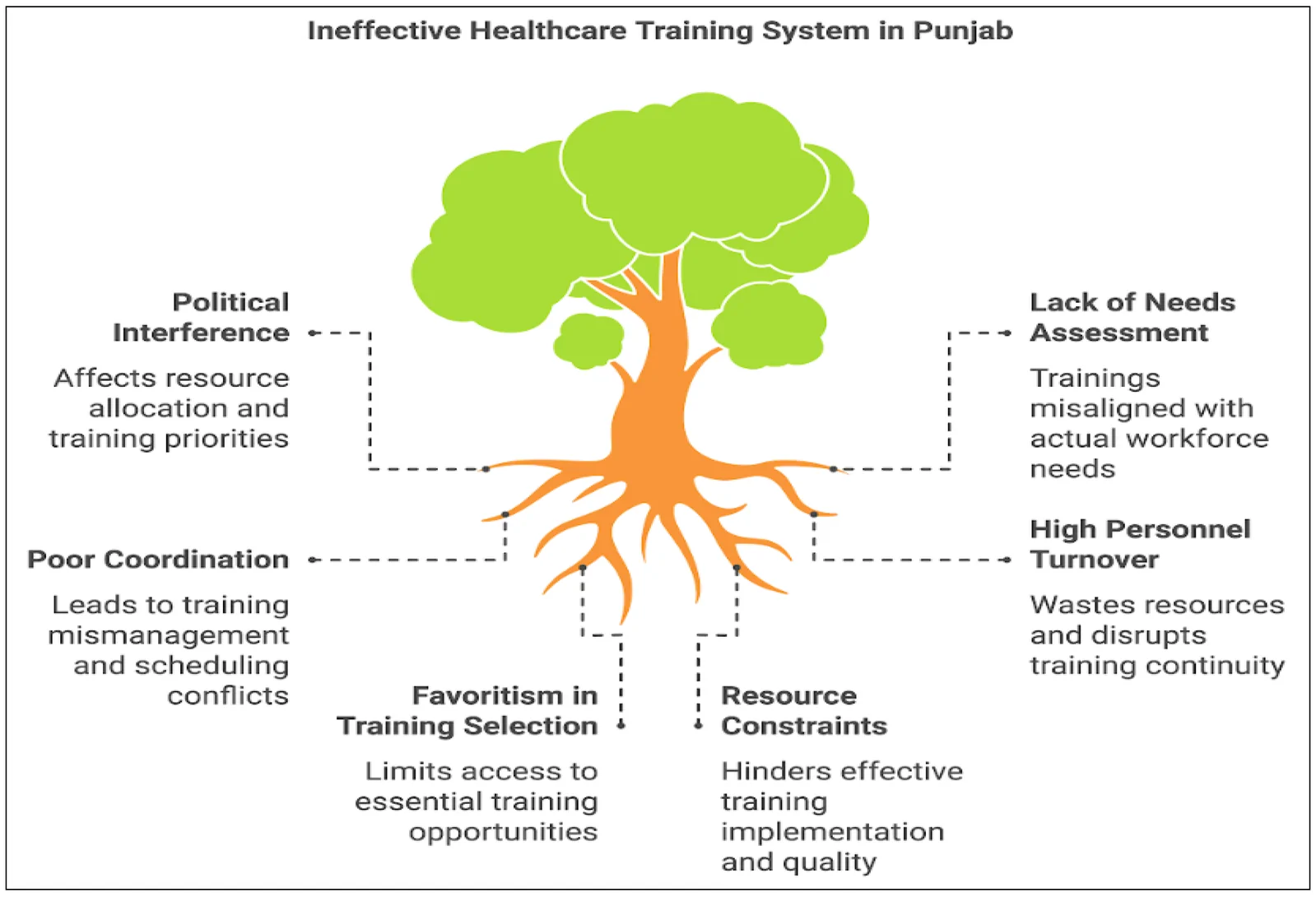
Systemic factors underlying an ineffective healthcare training system in Punjab.

The findings across the four capacity levels converge on six interrelated factors that constrain the effectiveness of in-service training in Punjab (see Figure 2). First, the absence of a formal needs-assessment mechanism means that training priorities are determined administratively rather than by systematically identified requirements. Second, political influence shapes both training priorities and resource allocation and extends to participant selection, where nomination practices were described as favoring particular individuals. Third, coordination between the department, vertical programs, and development partners is weak, resulting in duplication and scheduling conflicts. Fourth, resource constraints limit trainer quality and delay the release of funds. Fifth, the frequent transfer of personnel dissipates institutional capability. Sixth, the lack of robust evaluation leaves the system without feedback on what works.

Political interference and favoritism in participant selection were reported by stakeholders across cadres and regions and are presented here as participant perception; they were not independently verified in this study.

## 4. Training Framework for Healthcare Providers

**Figure 3:**
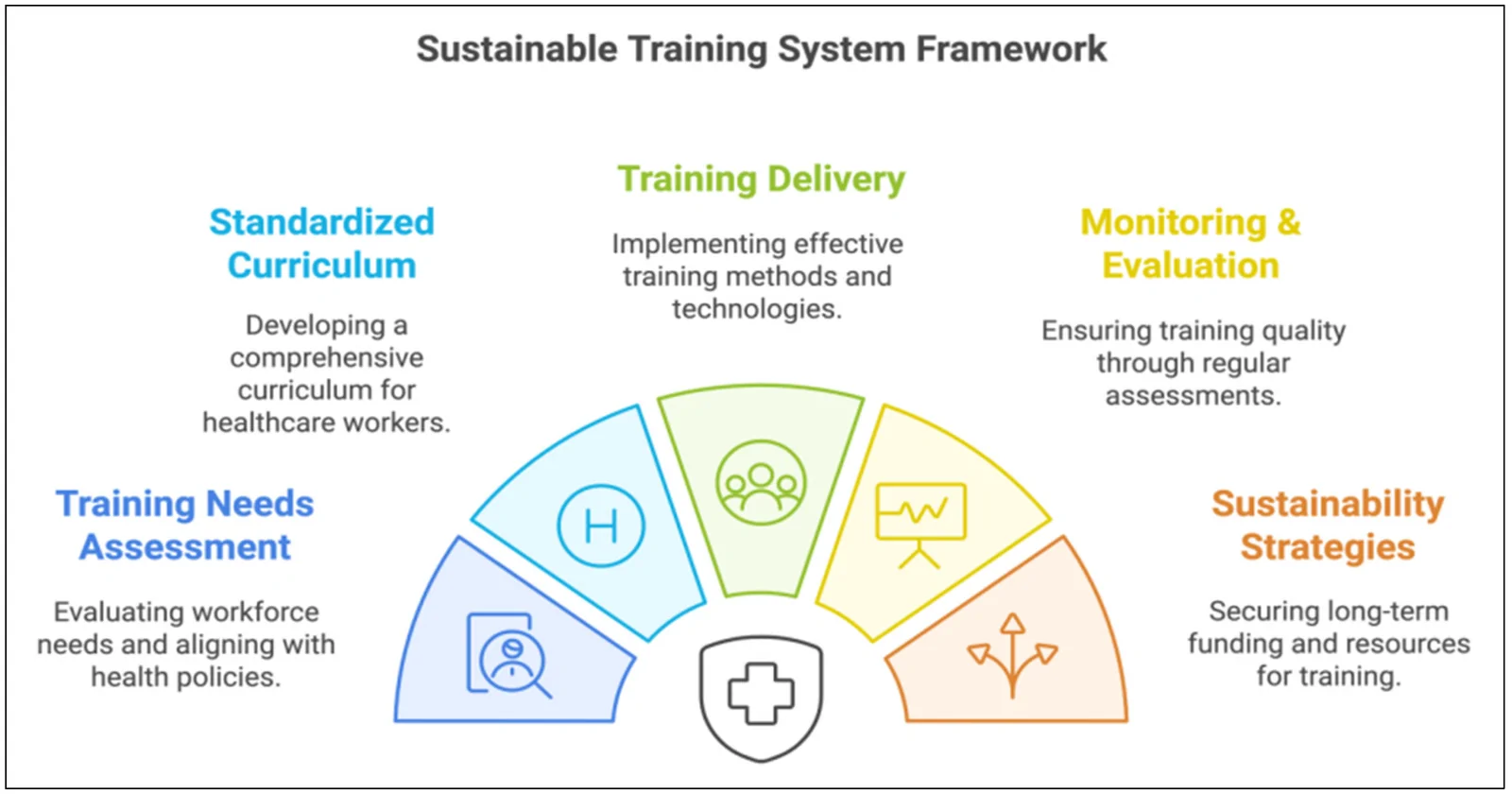
Sustainable training framework of in-service health care workers.

Based on the findings presented above, a training framework was constructed by integrating those findings with national and provincial policy requirements, international guidance on health workforce training, and established models of curriculum development and training evaluation. Developing a sustainable training system for the P&SHD, especially in a resource-limited context, requires a multi-pronged approach that addresses governance, educational design, delivery, evaluation, and financing together rather than in isolation. The proposed framework comprises five sequential components (see Figure 3). Training needs assessment ensures that the training addresses relevant and prioritized requirements and aligns with health policy. Standardized curriculum establishes a defined process for curriculum development, module creation, and trainer selection that establishes consistency and maintains high-quality standards across districts. Training delivery specifies effective methods and technologies, target audiences, timelines, and costing. Monitoring & evaluation provides for assessment of training quality and outcomes, and generates the feedback on which ongoing improvement depends. Sustainability strategies secure long-term funding and resources through advocacy for a dedicated training budget line, exploration of alternative funding sources, and optimization of available resources (see Figure 3). Quality assurance through continuing professional development and accreditation operates across all five components rather than as a discrete stage.

The framework draws on five established models, each contributing to a distinct dimension. Hennessy–Hicks [17] supplies the method for needs assessment, providing a systematic basis for identifying training requirements in place of the administratively determined prioritization documented here. Kern’s six-step approach [18] provides the overall educational architecture, with Bloom’s taxonomy [20] operating within it at the level of learning-objective specification. Kirkpatrick [21] supplies the internal structure of the monitoring and evaluation component, defining the levels at which outcomes are assessed. TRAIN [22] informs the sustainability component, specifically the development of a trainer cadre able to cascade capability without continued reliance on external expertise. Each of these models addresses one dimension and leaves the others outside its scope: Kern specifies curriculum development but not governance or financing; Kirkpatrick specifies evaluation but not needs assessment or implementation. The deficiencies identified in Punjab are not confined to any one dimension. The contribution claimed here is therefore not the derivation of new components but the specification of their interdependence within a single operational framework, derived from province-wide empirical assessment and specified for the governance and financing conditions of a decentralized public health system.

The derivation of each component from the study evidence is set out in Table 4, which states the deficiency the component addresses, the supporting findings from the institutional survey, the qualitative strand and the documentary review, and the established model contributing to its design.

The following section outlines key elements contributing to developing a comprehensive training framework.

### 4.1. Training Needs Assessment

A sustainable and effective training framework requires a thorough evaluation of the healthcare workforce’s specific training needs. This evaluation should incorporate global best practices aligned with national health policies and address emerging healthcare challenges [27]. Understanding the unique labor market dynamics of the country, including the supply of, demand for, and distribution of healthcare workers, as well as the economic factors that influence healthcare workers’ career choices, also enables the development of targeted policy responses [28]. The assessment should also examine existing training programs and identify areas needing improvement. This phase also examines available infrastructure and resources, including physical facilities, qualified trainers, and training budget allocations [24]. The needs assessment should evaluate current workforce competencies, future skill requirements, alignment with government priorities and financing mechanisms, and the availability of infrastructure, qualified trainers, training materials, and institutional resources necessary for effective implementation.

Training needs assessment (TNA) is a critical tool for identifying the learning and development areas that the healthcare department must address to overcome performance gaps and achieve quality healthcare outcomes [29]. TNA systematically identifies gaps between the current and desired levels of knowledge, skills, and abilities that healthcare workers require to perform effectively. Addressing these gaps through well-designed training programs can close these gaps, enhance workforce capabilities, and improve service delivery [29].

**Figure 4:**
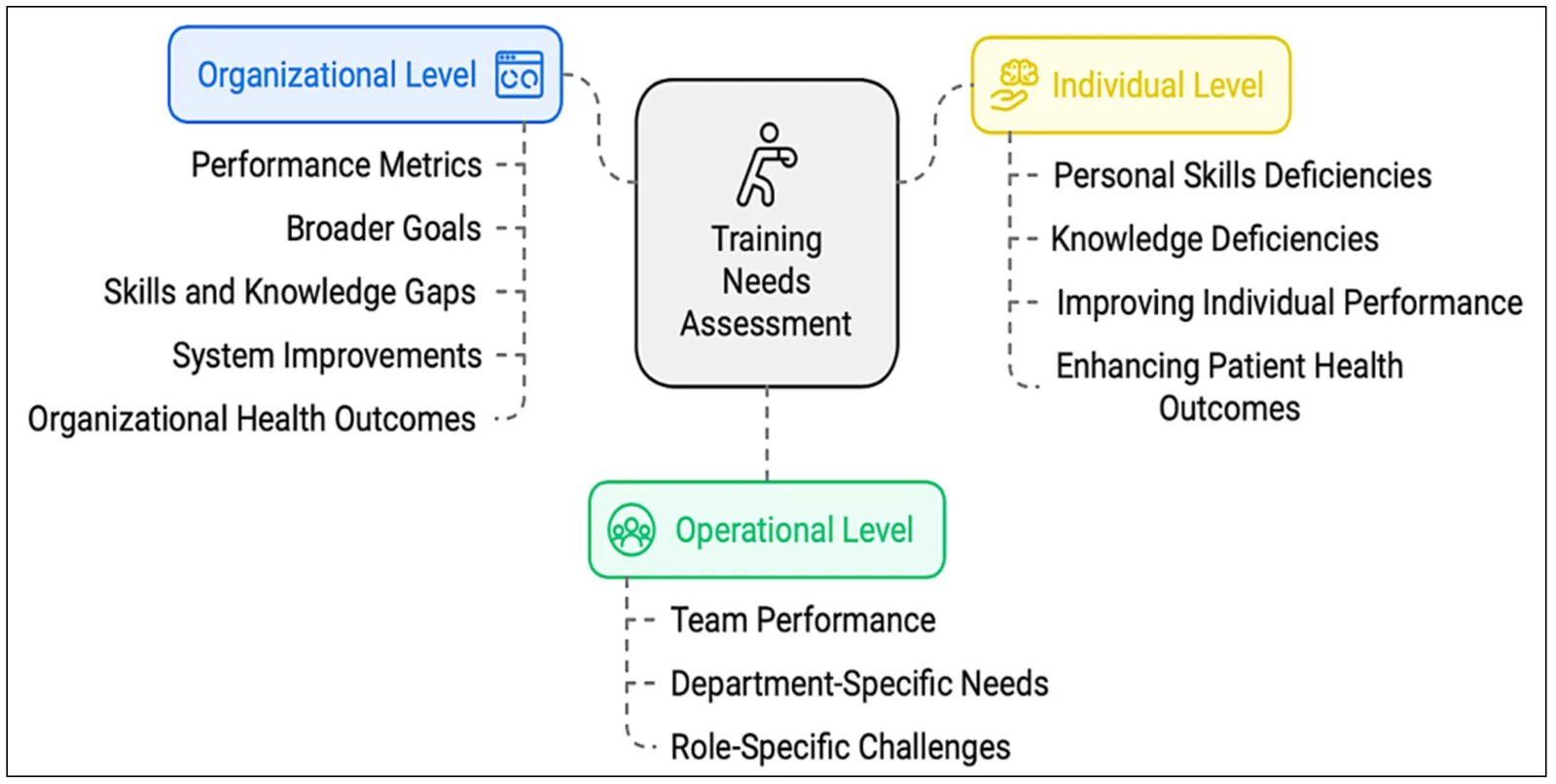
Training needs assessment at organizational, operational and individual levels.

The training needs assessment can be done at three levels:

1. The organizational level focuses on performance metrics and broader goals to determine the training required to meet the Health Department’s objectives. It identifies skills and knowledge gaps and training needs to improve organizational performance, system improvements, and organizational-level health outcomes;
2. Operational or job-role level needs assessment identifies specific training needs to improve a team, department, or particular group of healthcare workforce to address role-specific challenges and enhance team performance;
3. The individual level targets the training needs of specific healthcare workers, enabling tailored interventions to address personal skills or knowledge deficiencies required to improve individual performance and patient health outcomes [30] (see Figure 4).

A successful TNA aligns with best practices, beginning with clearly defined desired outcomes. It underscores identifying the activities required to achieve these outcomes before determining specific training initiatives. Effective TNA also depends on advanced stakeholder management, involving key groups such as the health workforce, patients or service users, trainers responsible for designing and delivering the program, and internal sponsors funding the training program. Addressing stakeholders’ expectations is crucial to the analysis and the program’s success [17].

Using integrated approaches, such as combining technical skill development with practical applications, ensures that training directly contributes to improved healthcare delivery [17,30]. By aligning TNA with these principles, healthcare departments can create targeted, impactful training programs that enhance workforce efficiency and achieve sustainable quality improvements in healthcare.

### 4.2. Standardized Curriculum

**Figure 5:**
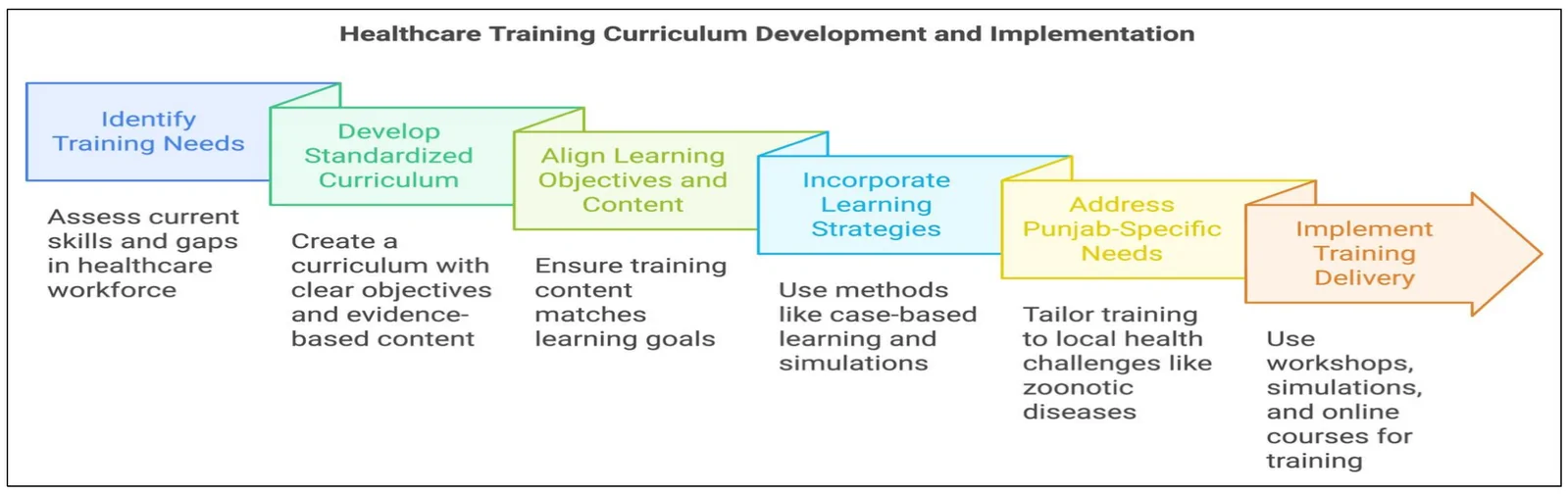
Components of standardized training curriculum.

A standardized curriculum addressing the identified training needs was developed, ensuring clear learning objectives, evidence-based content, and diverse teaching methodologies [18] (see Figure 5).

Clear, measurable, and performance-based learning objectives based on Bloom’s Taxonomy were integrated into the training framework [31]. Bloom’s Taxonomy provides a structured framework, dividing objectives into six categories: Knowledge, Comprehension, Application, Analysis, Synthesis, and Evaluation [20]. This classification guides curriculum developers in designing comprehensive and targeted training experiences. We also incorporated adult learning principles, emphasizing relevance, participation, repetition, feedback, and applied learning to strengthen competency acquisition and retention among healthcare workers [32].

Learning objectives and content must be developed simultaneously to ensure alignment. Training content needs to be evidence-based, relevant to healthcare settings, and designed to achieve specific outcomes [33]. It is also crucial to incorporate training on the specific needs of Punjab, such as zoonotic disease management, using a One Health approach, emphasizing collaboration between human and animal health sectors [23,34]. A sustainable training system must also be based upon a competency-based approach, catering to all levels of healthcare workers, from ambulance drivers to doctors, ensuring that all staff members possess the necessary knowledge and skills to deliver high-quality care [35].

The literature further emphasizes that the curriculum should be comprehensive and up-to-date, encompassing essential areas like:

- UHC and primary healthcare strengthening [36,37]
- Health system management and workforce leadership [2]
- Community engagement and population health approaches [37,38,39]
- Digital health, health information systems, and data-informed decision-making [37]
- Context-specific health priorities, including zoonotic disease management and One Health approaches [23,34]
- Competency-based training across clinical, administrative, and public health functions [13,37]

These domains were prioritized to address the gaps identified through stakeholder consultations, particularly the limited focus on managerial competencies, digital health systems, standardized clinical training, and interdisciplinary workforce development within Punjab’s healthcare system.

### 4.3. Training Delivery

The next step for developing a sustainable and effective training framework for healthcare workers is implementing the training program using various effective delivery methods. These may include case-based learning using patient scenarios to encourage problem-solving; simulation training to build clinical competence; interactive sessions for peer learning and discussion; and technology-based approaches such as e-learning modules, videos, and virtual reality to provide immersive learning experiences [40].

**Figure 6:**
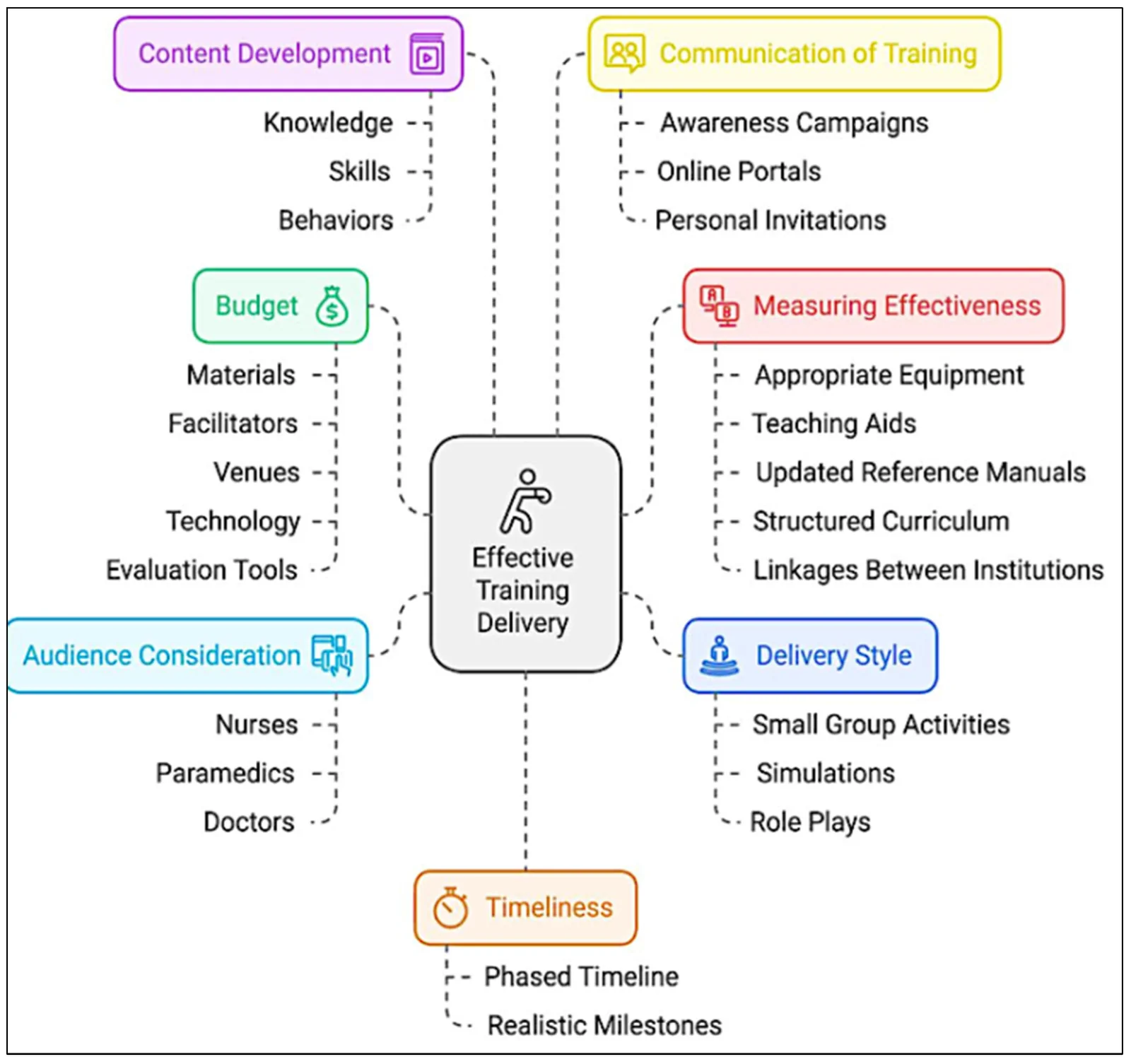
Components of effective training delivery.

The training implementation plan ensures the effective delivery of in-service healthcare workforce training by addressing critical aspects such as training content and approaches, cost, target audience, timeline, and communication (see Figure 6). The literature on best practices emphasizes that training content and approaches must be designed and implemented in a culturally relevant manner, ensuring sensitivity to learners’ needs [13,33,37,41,42]. Additionally, continuing capacity building and structured supervision are essential for providing ongoing support and ensuring sustainability [43].

A detailed budget outlines expenses for materials, facilitators, venues, technology, and evaluation tools, with cost-effective measures like online modules or partnerships to optimize resources [42]. Moreover, effective training delivery requires appropriate equipment, teaching aids, updated reference manuals, and a well-structured curriculum [43,44]. Equally important are strong linkages between pre-service and in-service training institutions to ensure the seamless implementation of training programs, fostering continuity and alignment in healthcare workforce development.

### 4.4. Monitoring and Evaluation of Trainings

A robust M&E system is another main component for developing a sustainable and effective training framework. The M&E system should include clear indicators, data collection mechanisms, and feedback channels to ensure training quality and inform improvement efforts [17,45]. The training system should also focus on building data management, analytical, and planning capacity to develop evidence-based human resource for health (HRH) policies and strategies [21].

**Figure 7:**
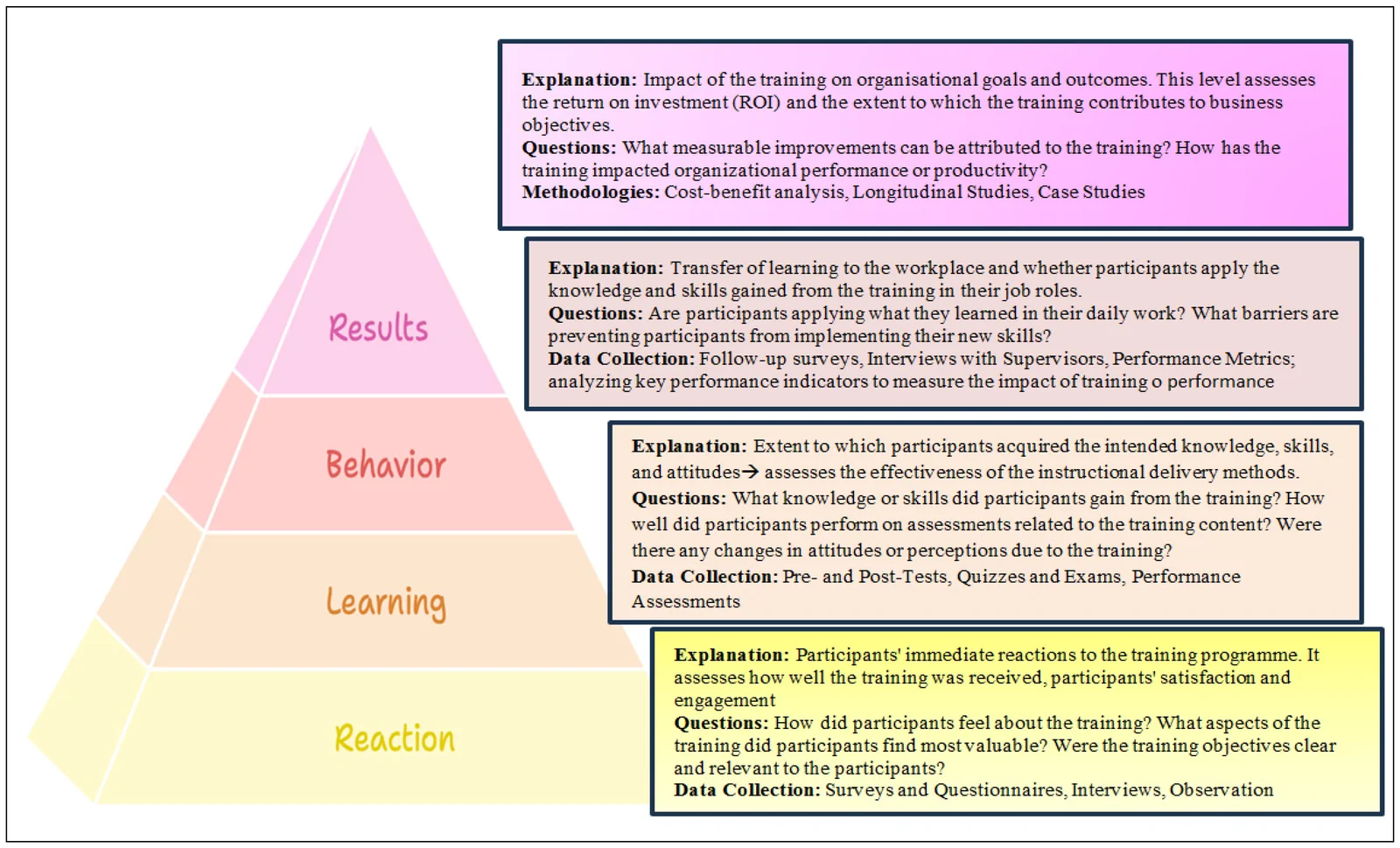
Kirkpatrick levels of evaluation (source: [21]).

Various evaluation frameworks are used globally for evaluating the effectiveness of training, for example, the Context-Input-Process-Product (CIPP) evaluation model [45], Kirkpatrick evaluation model [21,46], and Outcome-based Evaluation model (OBE) [47]. Kirkpatrick’s model was selected because it provides a structured approach for evaluating training reactions, learning, behavior change, and organizational outcomes [46,48,49,50]. The model categorizes four levels at which training can be evaluated: Reaction, Learning, Behavior, and Results (see Figure 7).

Level 1 of the Kirkpatrick Model measures the relevance and engagement of the training by assessing the reaction of the participants, such as their overall impression and feelings about the training [46]. It usually measures participants’ satisfaction with the training. It provides valuable insights into the quality of training materials, educators, session design, feedback mechanisms, and interactivity [46,50]. The second level of the model assesses the knowledge and skills participants acquired during the sessions. through paper or digital learning assessments [21,50]. Level 3 of the Kirkpatrick Model focuses on behavior change, assessing the application of knowledge and skills in the participant’s work environment [21]. Though measuring participants’ behavior is challenging, it can be evaluated by reviewing participants’ routine performance or metrics relevant to their roles [51]. The final level measures the training’s overall impact on operational performance and clinical outcomes [21,51]. The model highlights how the training contributes to broader organizational goals and healthcare quality improvements [21,22,23,24,25,26,27,28,29,30,31,32,33,34,35,36,37,38,39,40,41,42,43,44,45,46,47,48,49,50,51].

#### Key Outcomes and Measurable Indicators

Within the context of the Kirkpatrick Model, the key outcomes and measurable indicators for the M&E framework [21,22,23,24,25,26,27,28,29,30,31,32,33,34,35,36,37,38,39,40,41,42,43,44,45,46,47,48,49,50,51] are given in Table 5.

### 4.5. Sustainability Strategies

The training framework also requires a comprehensive sustainability plan that includes securing stable funding for the training system. This can involve advocating for a dedicated budget line item for in-service training [24].

Literature indicates that the Training of Trainer (TOT) is a key predictor of the sustainability of the training framework as it builds a cadre of skilled trainers who can cascade knowledge and skills to others within the system [22]. By empowering local trainers, TOT reduces dependency on external expertise, ensures continuity of training, and promotes institutional capacity-building.

**Figure 8:**
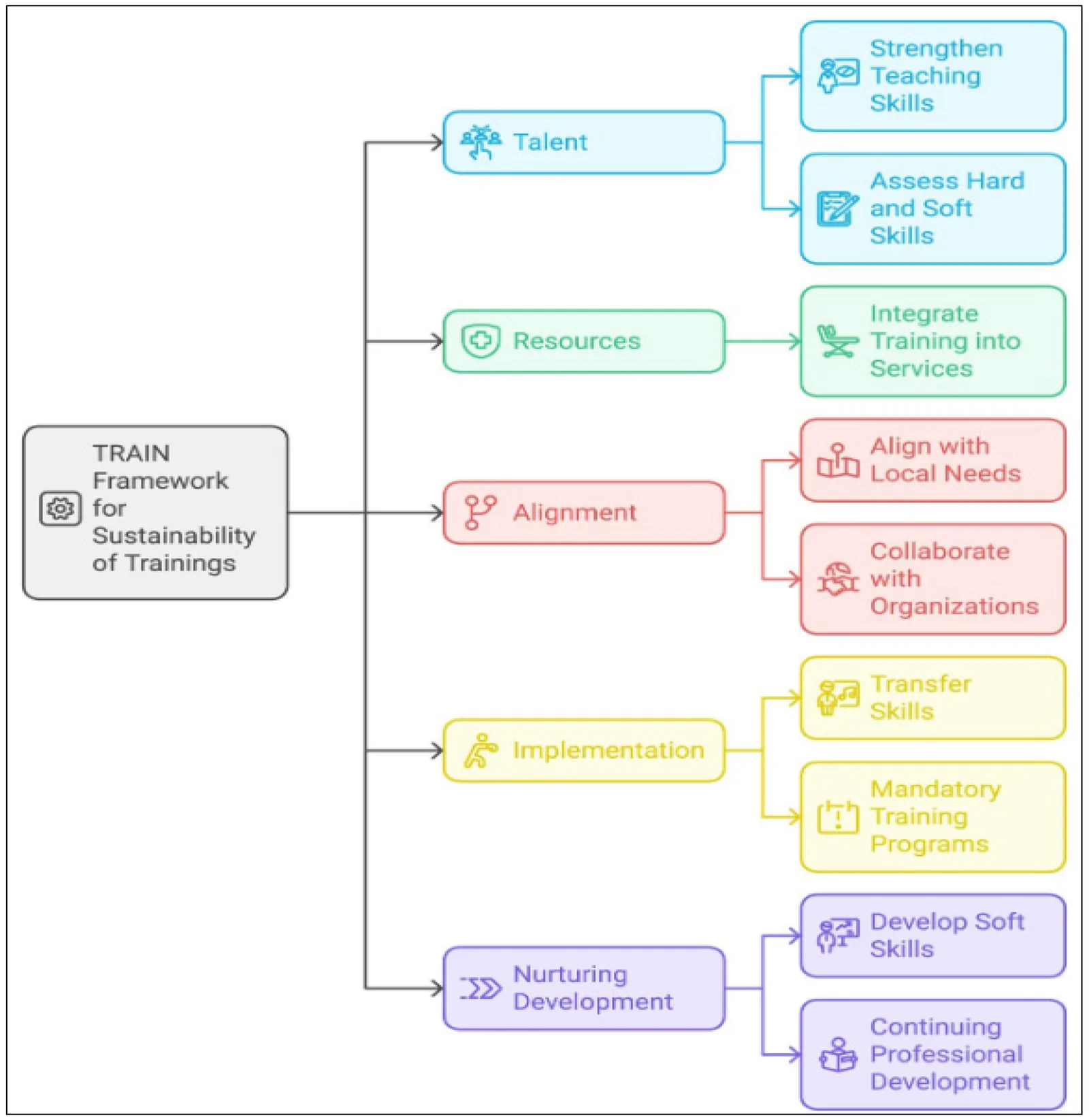
Components of TRAIN framework for sustainability of TOT.

The conceptual framework of TRAIN is used for TOT interventions [22]. The TRAIN framework focuses on developing sustainable training systems by addressing key challenges, strengthening ownership, and ensuring alignment with local contexts and professional goals. The key components of the framework include talent in the form of teaching skills, resource integration, alignment, implementation, and nurture and development [22,52,53] (see Figure 8).

### 4.6. Ensure Quality Through CPD and Accreditation

Implementation of CPD needs to be incorporated into an accreditation system with defined standards and certification processes [15]. This system helps to maintain program quality and recognizes the accomplishments of participants. Linking training outcomes to performance evaluations emphasizes the value of CPD and encourages active participation [26]. World best practices on training healthcare forces also emphasize prioritizing the CPD of in-country trainers and advocate for improvements in continuing education and professional development systems for all healthcare workers [24].

## 5. Discussion

Based on the data collected from DHDCs across Punjab and insights from various stakeholders, several critical challenges have been identified in the province’s healthcare training landscape. These include the absence of a uniform implementation plan, undefined roles and responsibilities, outdated policy documents, lack of a centralized governing body, weak coordination between stakeholders (including DHDCs and PHDC), job insecurity, low motivation among healthcare providers, and political influences in the decision-making process. Notably, these challenges coexist with substantial physical training capacity: nearly all responding DHDCs reported dedicated training halls, internet connectivity, and audiovisual equipment. This divergence indicates that infrastructure is not the binding constraint on training effectiveness, and that continued capital investment, in the absence of governance and evaluation reform, would be unlikely to alter the outcomes sought.

Regarding training capacity, the study highlights multiple barriers impeding the competency development of healthcare providers. These include the absence of a structured needs-assessment mechanism, financial constraints, a shortage of technically competent master trainers, unequal training opportunities, a lack of a standardized training system, limited focus on interpersonal and soft-skills training, and an inadequate M&E system for assessing training effectiveness. These challenges indicate a significant gap between Punjab’s current training framework and the guidelines of NHSP DLI 10, which emphasize integrating CPD into performance management systems, ensuring equitable resource allocation, and standardizing training delivery mechanisms [25]. To address these gaps, a sustainable and effective training system aligned with NHSP has been conceptualized using Kern’s approach [18]. The proposed framework aims to establish a cohesive structure that fosters collaboration, addresses systemic barriers, and improves CPD implementation for healthcare managers and professionals. The findings are consistent with previous studies from low- and middle-income countries demonstrating that fragmented governance structures, inconsistent training standards, and inadequate monitoring systems reduce the effectiveness of continuing professional development initiatives. Similar barriers have been reported in decentralized health systems in South Asia and sub-Saharan Africa, particularly regarding workforce retention, training equity, and institutional coordination [6,24,27,28,44,54,55,56].

The study also identified an apparent contradiction that proved analytically informative. All responding DHDCs reported delivering CPD to every cadre, while stakeholders described access as inequitable and some professionals as repeatedly trained while others remained under-skilled. These findings are reconcilable: institutional provision is universal, individual access is not, and the binding constraint lies in participant selection rather than in training supply. This distinction matters for what a training framework can achieve. Standardizing curricula and evaluation address the quality of training delivered to those who attend; it does not determine who attends. Transparent, criteria-based selection with individual training records is included in the proposed framework for this reason, but selection practices reported to be shaped by favoritism and political consideration are ultimately governance questions rather than educational ones.

A growing body of literature advocates for a comprehensive training system framework rather than ad hoc or isolated initiatives [55,57]. Best practices for healthcare training emphasize transitioning from compliance-driven approaches to capacity-building strategies that ensure sustainability and effectiveness [6,57]. Internationally, training models prioritize context-based and competency-driven approaches, focusing on skill development, authentic assessment methods, and continuous feedback [6,57,58,59].

International approaches differ from Punjab’s arrangement principally in where responsibility for participation is located. In an assessment of fifteen European countries, seven required all physicians to participate in CPD as a condition of licensure, and in most the body overseeing these requirements was the same body regulating the right to practice [60]. Where participation is a licensure condition, the practitioner rather than the employer holds responsibility for maintaining a record of activity, which relocates the selection decision away from the actor whose discretion participants in this study identified as the source of inequity. Mechanisms vary across Europe in ways reflecting differing regulatory traditions, with some countries relying on continuing medical education alone and others incorporating peer review, and with substantial divergence in monitoring and enforcement [61]. That divergence carries a caution relevant to Punjab: in several countries, participation is formally mandatory but not consistently enforced [60], indicating that a requirement without an enforcement mechanism changes little in practice. Effective decentralized systems also share features absent in Punjab, notably accreditation of training providers, a central record of completed activity, and mutual recognition of credit across jurisdictions [60]. These comparisons should be read with the difference in cadre scope in mind, since the European evidence concerns physicians whereas the present framework spans all cadres.

Sustainability in healthcare training requires a long-term vision, adequate financial investment, and institutional commitment. Secure and predictable financing is essential, necessitating increased government allocations, innovative financing mechanisms, and strategic donor support [62,63]. Additionally, building local capacity by developing in-country trainers, mentors, and training institutions ensures sustainability, reduces reliance on external experts, and ensures that training programs remain responsive to local needs [56,58,64].

The standardized training framework proposed in this study offers the P&SHD a roadmap to implement a comprehensive training and support program beyond mandatory courses and annual appraisals. The initiative could be introduced as a “competence certificate” or “care certificate”, emphasizing CPD and quality enhancement in healthcare. The training model follows the human capital investment approach to enhance professional competencies, job satisfaction, and workforce retention [65].

Continuous professional education, including in-service training and refresher courses, is crucial to ensuring healthcare workers remain competent. Research suggests that training programs should address knowledge and skill gaps while adapting to emerging healthcare challenges, particularly in low-income and middle-income countries (LMICs), to maximize resource utilization [66,67]. Promoting a culture of lifelong learning, facilitating peer learning networks, and encouraging participation in professional development activities may strengthen workforce capabilities [68,69].

Supportive supervision is another critical component for ensuring the quality and sustainability of healthcare training. Studies indicate that regular on-site mentoring, performance feedback, and structured problem-solving discussions significantly improve healthcare workers’ ability to apply newly acquired skills effectively [66,67]. Additionally, a robust M&E system is essential for tracking progress, assessing impact, and continuously refining training programs [18]. Ensuring inclusivity and accessibility through interdepartmental coordination and culturally relevant training materials further strengthens the framework.

International best practices also emphasize integration of cost-effective simulation-based and digital training tools to improve knowledge retention and overcome barriers related to geographical constraints [66,67]. Moreover, successful training programs involve collaborations with government agencies, academic institutions, and professional organizations to ensure alignment with healthcare priorities and sustainability [70]. Strengthening these partnerships may support capacity development and assist healthcare managers and supervisors to identify workforce challenges effectively [66]. The framework proposed in this study extends beyond conventional training delivery models by integrating governance, workforce planning, competency-based curriculum development, monitoring systems, and institutional sustainability into a unified approach to health systems.

Standardization and centralization are proposed here as responses to documented fragmentation, but both carry costs that warrant acknowledgement. Concentrating coordination in the PHDC would address fragmentation at the expense of district responsiveness, and districts differ materially in disease burden, workforce composition, and infrastructure. Standardization improves consistency while potentially serving cadres unevenly, since the requirements of a medical officer, a vaccinator, and a district administrator overlap little, and participants identified the absence of cadre-differentiated content, particularly managerial training, as a current deficiency. Digital delivery presents a similar tension: it could reduce travel burden and generate the training record the system lacks, but the internet availability reported here is an institutional measure that says nothing about connectivity, device access, digital literacy, or protected learning time for frontline workers, particularly outreach cadres in underprivileged districts. The framework attempts to reconcile these tensions through a common core with district- and cadre-adaptable modules and blended rather than wholly digital delivery, though whether that balance is workable is among the questions pilot implementation would need to answer.

### 5.1. Strengths and Limitations

#### 5.1.1. Strengths

This study draws on near-complete institutional coverage of the province. The survey was distributed to all 31 DHDCs rather than to a sample, and 26 returned completed instruments, giving a picture of institutional training capacity across Punjab rather than an inference from a subset. Data collection was undertaken officially with the support of the P&SHD and the PHDC, which secured access to institutional information, budgetary arrangements, organisational functions, staffing histories and five-year training records that are not ordinarily available to external researchers, and supporting documentation was collected alongside the questionnaire returns.

The qualitative component is unusually broad for a study of this kind. 33 FGDs with 270 participants and 20 KIIs were conducted across 11 districts in three regions, spanning six workforce cadres and every level of governance from frontline outreach workers to the provincial secretariat, together with development partners and vertical programme directorates. Grouping participants by cadre, holding sessions in private on official premises with only participants and the research team present, and conducting discussions bilingually in Urdu and English according to participant preference were all intended to support candour and to allow participants to describe their experience in the language they found most natural.

The framework itself is grounded in this evidence rather than imported from another setting, and its derivation is documented: each component is traceable to the specific quantitative findings, qualitative themes, policy requirements and established models from which it was drawn, and components resting on weaker evidence are identified as such. The draft framework was subsequently reviewed and endorsed by the PHDC, the institution that would carry responsibility for its implementation, providing an assessment of its content and institutional relevance by the body best placed to judge both.

#### 5.1.2. Limitations

Much of the quantitative evidence rests on institutional self-report. Collection through official departmental channels and the accompanying supporting documentation reduce, but do not eliminate, the scope for reporting error, and institutions describing their own capacity to an assessment associated with their parent department may present that capacity favorably. This may partly explain the divergence between the high availability of infrastructure recorded in the survey and the inadequacy described in the qualitative accounts. Five centers did not return questionnaires, so the quantitative findings describe the 26 responding DHDCs rather than the full provincial set.

The study measures what the training system has and does, not how well it works. Institutional capacity and reported training activity are not measures of training quality or effectiveness, and the training needs identified are those perceived by healthcare workers and officials rather than deficits established by competency assessment, which was not undertaken. The design is also cross-sectional, describing the system at a single point and offering no basis for judging whether the deficiencies identified are worsening, stable or improving.

Qualitative participants were nominated by DHA offices against specifications supplied by the research team. This route was necessary to reach the required cadres across eleven districts, but it means individual selection lay outside the researchers’ control, and nominees may be more experienced, more available or more favorably disposed toward existing arrangements than the wider workforce. This bears particularly on the accounts of political influence over training prioritization and favoritism in participant selection: those accounts were given by individuals nominated through the same offices whose practices were under discussion, and are for that reason more likely to understate than overstate the problem. They are reported as participant perceptions and were not independently verified.

Finally, although the framework was reviewed and validated by the PHDC, it has not been implemented, or prospectively evaluated. Institutional endorsement establishes that the framework is judged relevant and appropriate by those who would operate it; it does not establish that its adoption would improve workforce competency, training quality, service delivery or patient outcomes. Those questions remain open and require prospective evaluation. The framework’s content specifications are in any case bound to Punjab’s administrative structure, cadre configuration and financing arrangements; its component architecture may be applicable to other decentralized systems, but this was not tested.

## 6. Conclusions

The study concludes that PHDC and DHDCs play a critical role in training healthcare workers but face significant challenges related to governance, financial constraints, and coordination inefficiencies. Strengthening these institutions requires addressing leadership instability, enhancing interdepartmental collaboration, ensuring sustainable funding, and optimizing administrative processes to improve training effectiveness.

Implementing a standardized centralized, and inclusive training framework is crucial for building a skilled and efficient healthcare workforce. Such a framework is intended to support consistency in training quality, equitable access to professional development opportunities, and alignment with healthcare system needs and by fostering sustainability and continuous improvement, it may contribute to enhanced healthcare service delivery. The framework has been reviewed and endorsed by the PHDC, which indicated an intention to pilot two of the training modules, but it has not been implemented or prospectively evaluated; whether it improves service delivery or patient outcomes cannot be determined from this study and requires pilot implementation followed by prospective evaluation at the behavior and result levels.

## Figures and Tables

**Table 1:** Category and number of stakeholder groups for each region, and number of participants for focus group discussion.

Stakeholders	South	Central	North	Total FGDs	Total Participants
Doctors (from BHU, RHC & from DHQH, THQH)	2	2	2	6	48
Paramedics (LHVs, Vaccinators/EPI technician, school health & nutrition supervisors, pharmacy technician/Medical Technician)	2	2	2	6	48
Nurses	2	2	2	6	48
Outreach Program (Sanitary Inspector, LHWs, CMWs, LHS)	2	2	2	6	60
Administrative/Managerial Staff	2	2	2	6	48
Developmental Partners/Donors (WHO, UNICEF, International Fund for TB and Lung Disease)		1		1	8
Heads of the following Vertical Programs PD Aids Control Program PD Hepatitis Control Program/PD Infection Control Program PD TB Control Program PD CDC & Epidemics Prevention and Control (EPC) Program PD Non-Communicable Disease (NCD) Program		1		1	5
				
				
				
				
				
				
Heads of the following Vertical Programs PD Program Immunization PD Integrated Reproductive Maternal Newborn, Child Health & Nutrition Program (IRMNCH) Representatives of Family Planning Program		1		1	5
				
				
				
Total	10	13	10	33	270

BHU: Basic Health Unit; RHC: Rural Health center; DHQH: District Head Quarter Hospital; THQH: Tehsil Head Quarter Hospital; LHV: Lady health visitor; EPI: Expanded Program for Immunization; LHW; Lady health worker; CMW: Community Midwife; LHS: Lady health supervisor; WHO: World Health Organization; UNICEF: United Nations International Children’s Emergency Fund; PD: Project director; TB: Tuberculosis In addition, 20 KIIs were conducted with senior stakeholders, including the heads of DHDCs and higher educational institutes, Chief Executive Officers of District Health Authorities (DHAs), and representatives from the P&SHD and PHDC (see Table 2). Nominations for all KIIs were issued by the P&SHD. The research team specified the required post or institutional role; the P&SHD then identified the individual occupying that post and arranged the interview.

**Table 3:** Functions performed by DHDCs (n = 26).

Functions Performed by DHDCs	Yes		No	
	f	%	f	%
HRH policy and strategy				
Development of policies, strategies, plans, and frameworks for HRH	5	19.2	21	80.8
Coordinate an inter-sectoral District health workforce agenda, facilitating permanent mechanisms of collaboration among different line Departments (Education, Finance, Labour, etc.), including the private sector	5	19.2	21	80.8
Facilitate appropriate linkages on HRH between the District and Tehsil Administration;	8	30.8	18	69.2
Budgetary and resource planning and mobilization for HRH, in collaboration with the Department of Finance and other relevant constituencies	4	15.4	22	84.6
Define job descriptions, working conditions, supervisory and performance appraisal mechanisms, reward systems, and career structures for health workers in collaboration with relevant civil service bodies	5	19.2	21	80.8
HRH data, information and evidence				
Collection and analysis of health workforce data (e.g., stock, distribution, production, etc.)	1	3.8	25	96.2
Collection and analysis of health labour market data (e.g., employment status, vacancies, etc.)	0	0.0	26	100.0
Strategic analysis and monitoring of health workforce trends (including national and international mobility)	0	0.0	26	100.0
Stewardship of a District agenda on HRH research, including publication and dissemination of good practices	1	3.8	25	96.2
HRH accreditation, education and training				
Set policies on regulation, accreditation, certification, and education in collaboration with professional councils and academic institutions	4	15.4	22	84.6
CPD/training for health workers; Doctors	26	100.0	0	0.0
CPD/training for health workers; Nursing staff	26	100.0	0	0.0
CPD/training for health workers; Paramedics	26	100.0	0	0.0
CPD/training for health workers; Midwives	26	100.0	0	0.0
CPD/training for health workers; others	26	100.0	0	0.0
HRH leadership, advocacy, and policy dialogue				
Build capacity for HRH policy and management	10	38.5	16	61.5
Advocate for HRH investments and health workers’ rights and working conditions	6	23.1	20	76.9
Contribute to the management of labour relations with health workers’ unions/representatives	5	19.2	21	80.8
HR administration and management for health workers				
Payroll, entitlements, and leave administration	3	11.5	23	88.5
Decisions on employment, transfer, promotion, and disciplinary measures for staff	2	7.7	24	92.3
Other functions	12	46.2	14	53.8

Abbreviations: DHDC: District Health Development Centre; HRH: Human Resource for Health; CPD: Continuing Professional Development; f: frequency; %: percentage.

**Table 4:** Derivation of the proposed training framework components from the study evidence.

Component	Deficiency Identified in This Study	Supporting Evidence	Model Informing Design
1. Training needs assessment	Training areas determined administratively rather than by assessed need	Officials acknowledged the absence of a formal needs assessment mechanism; training was reported as politically driven and influenced by international funding, with participants describing existing training as misaligned with on-the-ground requirements; DHDCs prioritized education and capacity building while overlooking managerial and soft skills	Hennessy–Hicks [17]; Kern steps 1–2 [18]
2. Standardized curriculum	No standardized modules, curricula, or criteria for selecting resource persons	Inconsistent training packages reported across districts; 61.5% of DHDCs had a trainer program; participants called for standardized training packages, clear SOPs, and improved modules; a key informant identified module and trainer quality as the principal technical deficiency	Kern steps 3–4 [18]; Bloom’s taxonomy [20]; competency-based framework [13]; MSDS [23]
3. Training delivery	Fragmented and uncoordinated delivery across departments, vertical programmes and development partners	Vertical programs funded through PC-1 budgets and development partners reported to conduct training independently, bypassing DHAs, PHDC and DHDCs; 19.2% of DHDCs coordinated an inter-sectoral workforce agenda and 30.8% facilitated district–tehsil linkages; 19 of 26 reported to multiple authorities; weak interdepartmental synergy and coordination failures with donors was described	Kern step 5 [18]; USAID in-service framework [24]
4. Monitoring and evaluation	Evaluation limited to attendance and satisfaction; no workforce information base	3.8% of DHDCs collected health workforce data; none collected labor market data or monitored workforce trends; 3.8% stewarded an HRH research agenda; considerable variability was reported in assessment mechanisms including pre- and post-tests and follow-up; participants called for regular M&E to assess training impact	Kirkpatrick [21]; Kern step 6 [18]; NHSP DLI 10 [25]
5. Sustainability strategies	Insecure and procedurally constrained financing; loss of trained personnel through transfer	15.4% of DHDCs participated in budgetary and resource planning; all reported budgetary constraints; training allowances met by donor-funded programs but not by the department; difficulty recruiting competent trainers attributed to inadequate financial incentives; budget release described as requiring extensive approvals; direct allocation to PHDC and DHDCs advocated by key officials; frequent transfers reported to dissipate institutional capability, with 53.8% of DHDCs having three or more project directors in five years	TRAIN [22]; Kern step 6 [18]
Cross-cutting: Quality assurance through CPD and accreditation	No accreditation system or defined quality standards	15.4% of DHDCs set policies on regulation, accreditation, certification and education in collaboration with professional councils and academic institutions; participants across FGDs identified a centralized accreditation system and intersectoral collaboration with teaching institutions as necessary to ensure quality and consistency	WONCA CPD standards [26]

**Table 5:** Key outcomes and measurable indicators for monitoring and evaluation.

	Indicator	Measurement	Timeline	Responsibility
Input Indicators: These indicators track the resources and processes required to deliver training programs effectively.				
Training Resources	Number of training materials (manuals, guides, digital content) developed and distributed.	Count of training materials distributed to participants (by type and format).	Quarterly.	Training Coordinators.
Trainer’s Capacity	Number of qualified trainers and facilitators available.	Total number of trainers certified or trained for the program.	Annually.	PD PHDC
Infrastructure	Availability of training facilities and equipment.	Checklists of facilities and equipment required for training (e.g., classrooms, audi–visual aids).	Bi-annually.	PD PHDC &Team.
Process Indicators: These indicators track the processes and activities involved in implementing the training program				
Training Delivery	Number of training sessions conducted.	Count of scheduled and delivered training sessions.	Monthly.	PD DHDC/Training Coordinators
Participant Engagement and Attendance	Training attendance rate using digital attendance system	Percentage of registered participants who attended the training sessions.	Real-Time	Training Coordinators/PHDC/DHDCs
Training Curriculum Adaptation	Frequency of curriculum updates or revisions based on emerging healthcare needs.	Number of curriculum revisions based on feedback or new healthcare standards.	Annually.	PD PHDC & Team
Stakeholder Engagement	Number of collaborative meetings with key stakeholders (government, NGOs, private sector).	Count of formal stakeholder meetings held.	Quarterly	PD PHDC
Output Indicators: These indicators track the immediate results or outputs of the training programs.				
Knowledge Acquisition	Percentage of participants passing post-training assessments using pre- and post-tests	Percentage of participants who score above a predetermined threshold in assessments.	Pre-test before training and post-test immediately after each training session	Trainers, PD DHDC.
Feedback mechanism	Percentage of participants satisfied with the training content, structure, and module	Feedback forms and surveys	Immediately post-training	Training Coordinators & Facilitators
Skills Application	Percentage of participants reporting improved job performance due to the training.	Self-reported data from post-training surveys or focus groups.	3–6 months after the training session.	PHDC M&E Team
Outcome Indicators: These indicators track the medium-term effects of the training on healthcare services and outcomes.				
Quality of Care	Improvement in healthcare service quality post-training (e.g., improved diagnosis rates).	Quality assessments (e.g., patient satisfaction surveys).	6–12 months after the training.	Technical Wing/AS Technical PSHD
Health Outcomes	Improvement in health outcomes (e.g., improved vaccination coverage).	Health data from health facility reports (e.g., vaccination rates).	Annually.	HISDU/DHIS Cell.
Impact Indicators: These indicators assess the long-term effects of the training program on the health system.				
Healthcare System Strengthening	Improvement in health system indicators (e.g., health workforce capacity).	Health system performance indicators (e.g., utilization of health services).	Every 2 years.	Technical wing/AS Tech
Sustainability of the Program	Percentage of costs covered by government, alternative funding sources	Proportion of the program budget covered by government, alternative sources or self-generation	Annually.	Technical Wing/AS Tech

## Data Availability

The raw data supporting the conclusions of this article will be made available by the authors on request.
